# Development of SiC/PVB Composite Powders for Selective Laser Sintering Additive Manufacturing of SiC

**DOI:** 10.3390/ma11102012

**Published:** 2018-10-17

**Authors:** Peng Zhou, Huilin Qi, Zhenye Zhu, Huang Qin, Hui Li, Chenglin Chu, Ming Yan

**Affiliations:** 1 Department of Materials Science and Engineering and Shenzhen Key Lab for Additive Manufacturing of Advanced Materialas, Southern University of Science and Technology, Nanshan District, Shenzhen 518055, China; zhoupeng85723@163.com (P.Z.); qihl@mail.sustc.edu.cn (H.Q.); qinhuang@stu.hit.edu.cn (H.Q.) lihui_knight@163.com (H.L.); 2 School of Materials Science and Engineering, Southeast University, Nanjing 210096, China; clchu@seu.edu.cn; 3 School of Materials Science and Engineering, Harbin Institute of Technology (Shenzhen), Shenzhen 518000, China; zhuzy@hit.edu.cn

**Keywords:** SiC, ceramic/polymer composite powder, 3D printing, ball milling, surface modification

## Abstract

Subsphaeroidal SiC/polymer composite granules with good flowability for additive manufacturing/3D printing of SiC were prepared by ball milling with surface modification using polyvinyl butyral (PVB). PVB adheres to the particle surface of SiC to form a crosslinked network structure and keeps them combined with each other into light aggregates. The effects of PVB on the shape, size, phase composition, distribution and flowability of the polymer-ceramic composite powder were investigated in detail. Results show that the composite powder material has good laser absorptivity at wavelengths of lower than 500 nm.

## 1. Introduction

Silicon carbide, SiC, has excellent physical and chemical properties, such as high mechanical performance, high thermal conductivity, low thermal expansion, and good wear, oxidation and corrosion resistance [1,2]. These properties make it an attractive candidate for a variety of industrial applications, such as machinery industry, aeronautic and aerospace engineering, automotive industry, and electronic and information engineering [1,3,4]. However, its industrial application has been limited so far, due to the related high production cost originating from its expensive mold manufacturing, sintering process at high temperature/pressure, and/or subsequent machining required [1,5,6]. Up to now, low temperature pressureless sintering and near-net and complex shaping have been challenging topics for SiC. In particular, conventional ceramic forming technology has great difficulty in fabricating ceramic parts with complex shapes [7]. 

As a new forming technique originated in the 20th century, additive manufacturing/3D printing is a versatile free-form technique characterized by its easy operation, high precision and near net shaping capability [8,9]. Selective laser sintering (SLS) is one of the mainstream 3D printing techniques, where parts are fabricated layer by layer using a laser without using a preform or a mold [10,11,12]; during processing using SLS, raw powder materials are normally not fully melted, which is different from selective laser melting where powders are generally fully remelted and then solidified [13]. Currently, SLS technology is widely studied and developed for additive manufacturing of polymers and metals, and various polymer and metal powders for 3D printing can be commercially purchased [10,14,15]. However, the dense ceramic parts fabricated by SLS have not yet been realized due to the high melting temperature, low or no plasticity and the low thermal shock resistance of ceramics [14]. 

To address this issue, researchers have developed two approaches to fabricate ceramic parts using SLS technology, i.e., direct SLS and indirect SLS. In the case of direct SLS processing, the as-sintered ceramic parts normally have low density and poor mechanical properties. In particular, thermal cracks in the ceramic parts tend to emerge due to the high thermal stresses resulting from SLS processing [16,17,18,19]. For indirect SLS processing, the polymers are used for a sacrificial binder phase. There are three steps for indirect SLS: (a) The first step is to select a suitable ceramic and polymer phase to prepare ceramic/polymer composite powders as the starting materials of indirect SLS; (b) the second step is to use a laser to melt the organic phase in the ceramic/polymer composite powder, and then the ceramic particles will be bonded by the binder and the green parts are prepared; (c) the final step for indirect SLS is to remove the binder and sinter the green part to increase its density and strength. Among these steps, the most important one lies in the production of polymer-ceramic composite powder agglomerates [14,20]. A great majority of the ceramic powders that are commercially purchased are severely agglomerated and present irregular morphology, and have poor flowability and are not suitable for 3D printing [21,22,23]. 

In this paper, the ball milling method was used to prepare SiC-polymer composite powders with good flowability and dispersity for indirect SLS processing. Polyvinyl butyral (PVB) was used as a binder to systematically investigate its effect on the surface modification of the SiC powders, and its effect on the shape, size, distribution and flowability of the polymer–ceramic composite powder agglomerates have been further discussed. Laser absorptivity of the composite powder was studied, as well to provide essential information for its SLS processing. 

## 2. Experimental Procedure

Commercially purchased SiC (mean particle size 1 μm, Weifang Kaihua Micro-powder Co., Ltd., Weifang, China) was used for the composite material development. In addition, polyvinyl butyral (PVB, average molecular weight of 40,000–70,000, Aladdin, Shanghai, China), polyvinylpyrrolidone (PVP), and anhydrous alcohol were used as a binder, dispersant and solvent, respectively; key information of the PVB material is listed in Table 1. 

The SiC powder and PVP were mixed together with anhydrous alcohol and then ball milled at 120 rpm for 12 h. For the ball-milling process, two different sizes (Ф 5 mm and Ф 3 mm) of zirconia balls at the weight ratio of 3:2 were used and different weight contents of PVB were added and sequentially ball-milled for 3 h at a rotation speed of 120 rpm. The milled suspension was dried at 50 °C using a drying oven. After drying, the SiC/PVB mixtures were mechanically granulated. The SiC/PVB composite powders obtained were sieved through a 120 mesh screen (i.e., powder size below ~120 μm). The preliminary spreading and forming tests of the SiC/PVB composite powders were carried out using a 3D printing machine (Concept Laser M2, Concept Laser GmbH, Lichtenfels, Germany). All tests were carried out at room temperature.

Scanning electron microscopy (SEM) was used to examine the morphology and microstructure of the granulated particles. The phase composition of the composite powders was identified using X-ray diffraction (XRD) using a Rigaku diffractometer model UltimaIV (Rigaku, Tokyo, Japan, Ni-flltered Cu Kα radiation; *λ* = 1.5406 Å). The X-ray tube was operated at 40 kV and 40 mA. The scanning range for the XRD diagrams was from 20° to 80° in 2*θ* angle with a step of 0.02° and scanning time of 1 s per step. Hausner ratio (HR) and Carr index were also measured to characterize the flowability of granules. The apparent density and tap density were measured according to the national standards of China, GB5061-85 and GB5162-85, respectively. The size of the agglomerates was measured using laser diffraction (Mastersizer Plus, Malvern, UK). The UV-Vis analysis of the SiC/PVB composite powders was tested using a UV/Vis/NIR Spectrometer (Lambda 950, Perkin Elmer, Waltham, MA, USA). 

## 3. Results and Discussion

**Figure 1:**
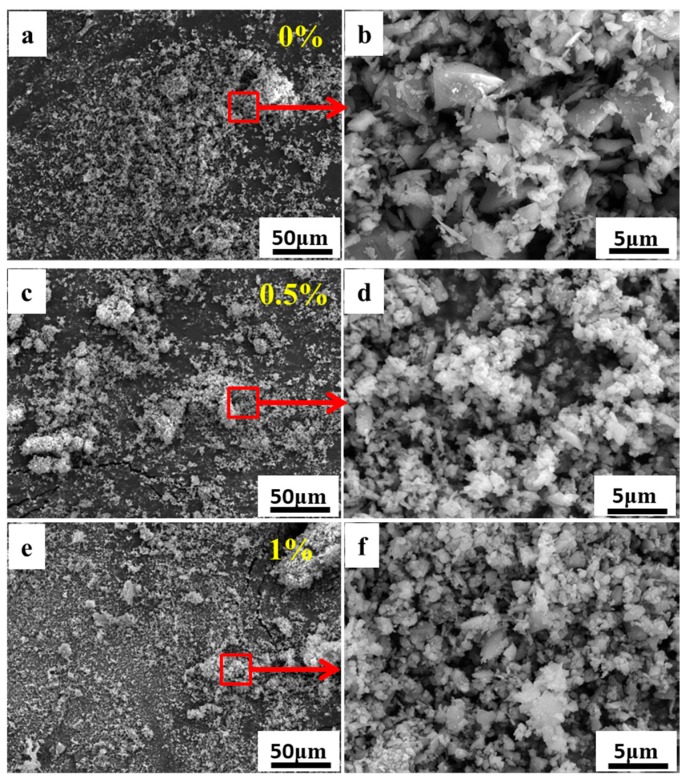
SEM images of SiC/PVB composite powders with different weight contents of the PVB binder. (**a**,**b**) for 0 wt. %; (**c**,**d**) for 0.5 wt. %; (**e**,**f**) for 1 wt. %.

**Figure 2:**
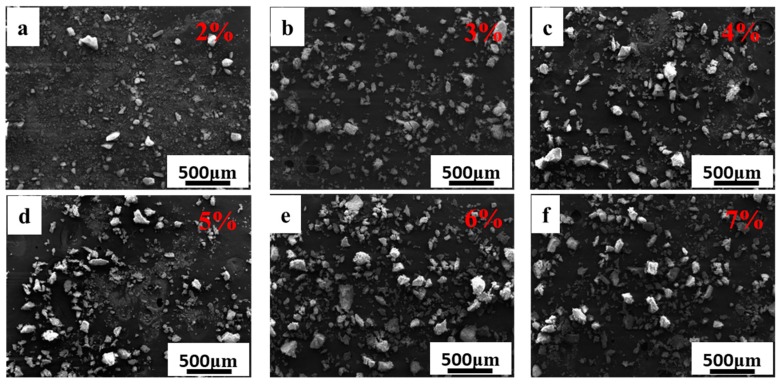
SEM images of SiC/PVB composite powders with the PVB binder contents in the range of 2 to 7 wt. %. (**a**–**f**) are 2 wt. %, 3 wt. %, 4 wt. %, 5 wt. %, 6 wt. % and 7 wt. %, respectively.

Figure 1 and Figure 2 show the SEM images of the granulated particles with different weight contents of PVB. It is noted that the binder can be dispersed homogeneously into the suspension during the ball milling. The binder adheres to the surface of the ceramic powders to form a crosslinked network structure around the particles [26], which leads to the single particles combining with each other into light aggregates. It should be noted here that the distance between the molecule chains of the PVB binder will be reduced owing to the drying process, and the absorption force between molecules should be enhanced. This is the reason why the crosslinked network occurs. As can be seen from Figure 1, the granulated particles are fluey due to the weak combining strength between the ceramic particles when the PVB content is less than 2 wt. %. Increment in the polymer content increases the size of the individual particles and the degree of agglomeration. With the increase of the PVB content, granulated particles show denser aggregates with a subsphaeroidal shape, as shown in Figure 2.

**Figure 3:**
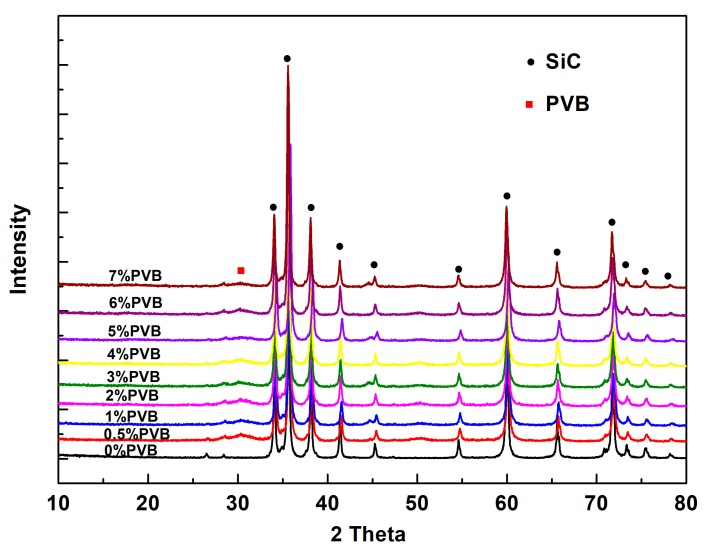
The XRD patterns of SiC/PVB composite powders with different PVB binder weight contents.

Figure 3 shows the XRD patterns of the SiC/PVB composite powders with different PVB binder contents. It can be seen that the characteristic peaks of SiC and a few diffuse diffraction peaks were detected. The diffuse diffraction peaks (e.g., those around 32°) are the characteristic peaks of amorphous substances, which indicates the existence of a PVB binder (which is an amorphous phase) in the composite powders. 

**Figure 4:**
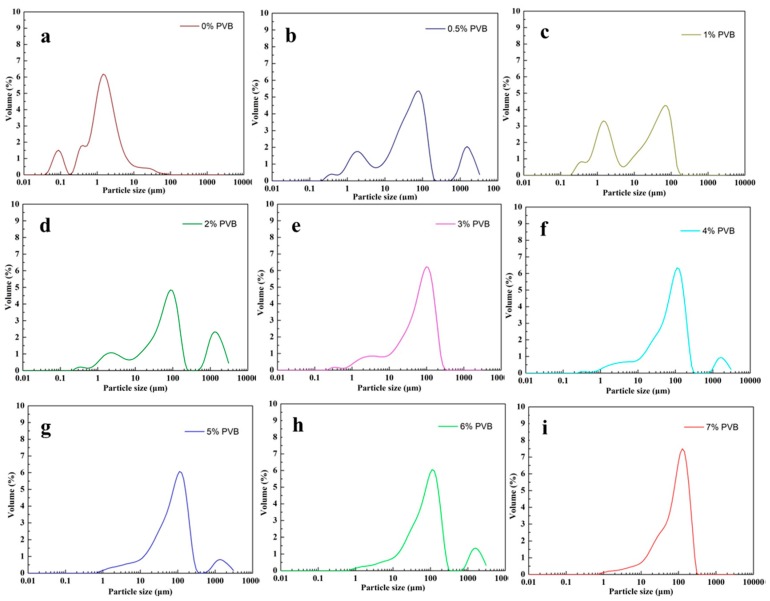
Size distributions of SiC/PVB composite powders with different contents of PVB binder.

Figure 4 shows the size distributions of the SiC/PVB composite powders with different contents of the PVB binder. It can be noted from Figure 4 that the size distribution of SiC/PVB aggregates is also influenced by the content of the binder. When the PVB content is less than 2 wt. %, the size distribution of the SiC/PVB granulated particles shows several peaks, as shown in Figure 4a–c. When the PVB content increases, the size distribution of the SiC/PVB granulated particles almost shows a monomodal distribution in a narrow range, particularly those shown in Figure 4e–i. 

**Figure 5:**
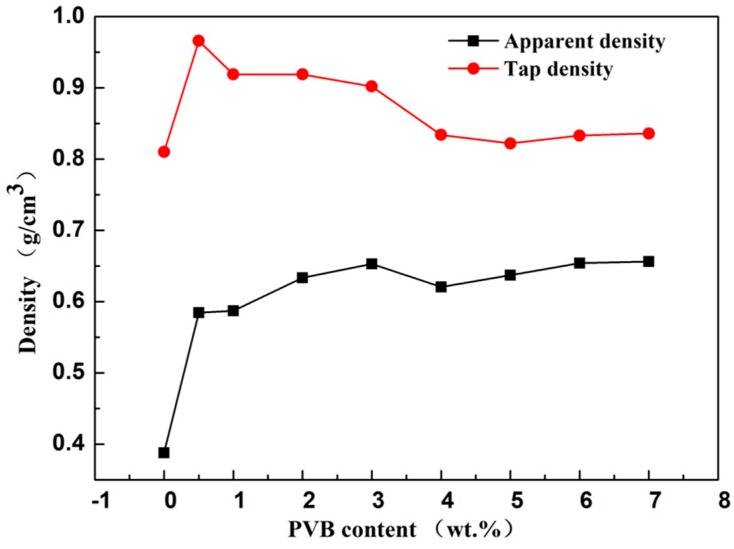
The apparent density and tap density of SiC/PVB composite powders with different PVB contents.

For testing and calculating the Carr index and Hausner ratio of the SiC/PVB composite powders, the apparent density and tap density were tested using the national standards of China. Figure 5 shows the apparent density and tap density results of SiC/PVB composite powders with different PVB contents. The apparent density increases with increasing binder contents until a transition occurs when the PVB content is 3 wt. %. It shows that, when the PVB content is more than 3 wt. %, the apparent density only slightly changes with the increasing PVB content. For the tap density, it will also increase evidently with increasing binder contents if the PVB content is less than 0.5 wt. %. Furthermore, the tap density decreases slightly with the increasing of the PVB content, when the PVB content is more than 0.5 wt. %. The highest tap density is 0.92 g/cm^3^ when the PVB content is 0.5 wt. %. It can be noted from Figure 5 that the heavy addition of the PVB does not have much influence on the apparent density and the tap density. 

It is well known that the flowability and filling property of aggregates can be represented by the apparent density [9]. The increasing PVB binder contents induce more solid particles into connection, which leads to an increase of aggregate size and decreases of Vander Waals forces between granulated particles within the SiC/PVB composite powder. Hence, the bigger aggregate tends to have better flowability and filling property. Furthermore, increasing the PVB binder contents is good for forming solid and dense aggregates, which can also increase the apparent density. Excessive binder, however, may form oversized aggregates and likely leads to larger and uneven porosity that is detrimental to the SLS of ceramics; meanwhile, it also decreases the tap density because bigger aggregates would lead to bigger void content [9].

**Figure 6:**
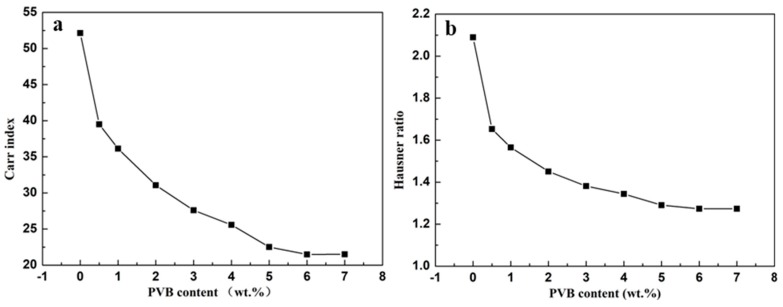
The Carr index (**a**) and Hausner ratio (**b**) of SiC/PVB composite powders with different PVB contents.

For the characterization of flowability of the powders, the Carr index and Hausner ratio are the important performance indices [27,28]. In order to give a precise description of the flowability of SiC/PVB composite powders with different contents of PVB binder, the Carr index and Hausner ratio are shown in Figure 6. Both the Carr index (Figure 6a) and Hausner ratio (Figure 6b) decrease with the rise of the PVB content. When the PVB content increases to a higher value, the heavy addition of PVB will not have much influence on the Carr index and Hausner ratio, just like the densities as measured above.

**Figure 7:**
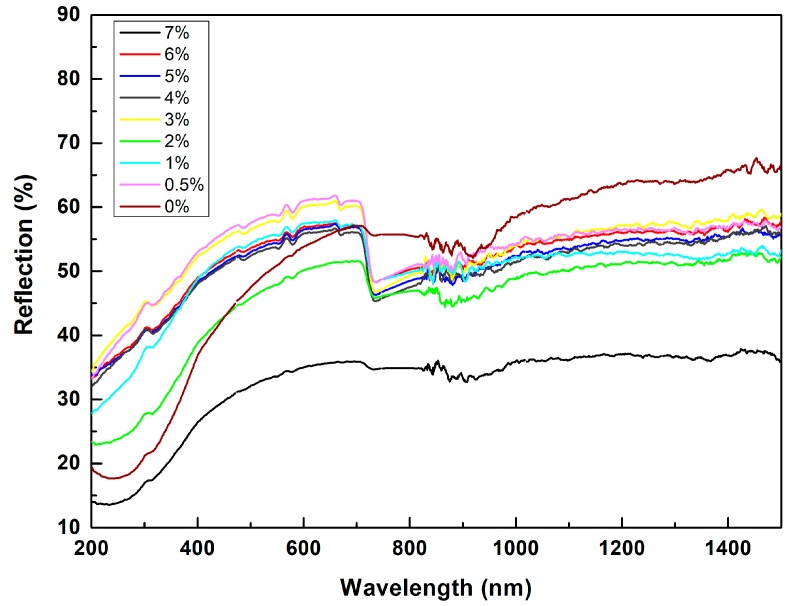
The UV-Vis analysis results of the SiC/PVB composite powders with different PVB contents.

For SLS processing, the energy for processing materials comes from the laser used, and from this perspective, an important requirement is that materials can absorb as much laser energy as possible at the specific wavelength of the laser being used (for the machine used in the present study, the wavelength of the laser is 1064 nm). Figure 7 shows the UV-Vis analysis results of the SiC/PVB composite powders with different PVB contents. As can be seen from Figure 7, the reflection of the SiC/PVB composite powders increases with the increasing of the wavelength when the wavelength is within a lower level (e.g., less than 500 nm). Furthermore, the reflection of the SiC/PVB composite powders basically remains unchanged when the wavelength is above 800 nm, except for the SiC/PVB composite powder without PVB addition. It can be noted from Figure 7 that the reflection of the SiC/PVB composite powders is lower than that of SiC/PVB composite powder with 0% PVB addition, at a wavelength of 1064 nm. This suggests that: (a) the addition of PVB is good for SLS of SiC/PVB composite powder from the laser absorptivity perspective, and (b) the best lasers for SLS processing of SiC/PVB composite powder are those with a wavelength of below 500 nm.

**Figure 8:**
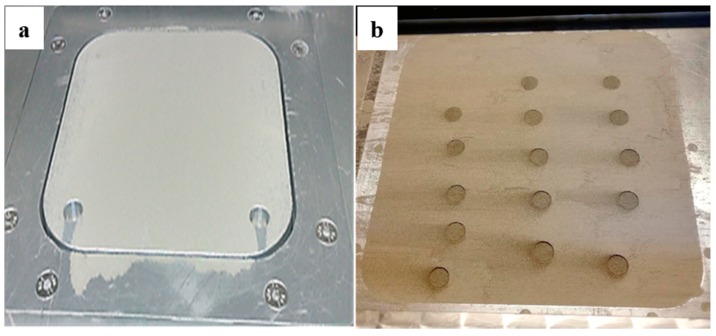
The typical spreading (**a**) and forming (**b**) tests of SiC/PVB composite powders with 3 wt. % binder addition using the 3D printing machine.

For SLS processing of ceramics, the first key aspect is that the powders should be able to spread out on the substrate of a 3D printing machine. To confirm this, the flowability of the SiC/PVB composite powders developed was tested, and the typical images of spreading and preliminary forming tests are shown in Figure 8a,b, respectively. It can be seen from Figure 8a that the powders are distributed uniformly on the powder bed by the recoater. This suggests that the SiC/PVB composite powders with a certain bind addition have a good flowability and forming performance. The circular areas in Figure 8b were the areas scanned using the laser. The as-sintered densities of the scanned areas (details omitted here) were tested, but the test results indicated that a simple touch by hand could break the scanned areas into pieces. This was mainly due to the high wavelength (~1064 nm) laser used for the SLS test. Further experiments based on using a lower wavelength, high power 3D printer are in progress, and the corresponding results will be presented in our future work.

## 4. Conclusions

Subsphaeroidal SiC/polymer composite granules with good flowability were prepared for SLS additive manufacturing/3D printing with surface modification by adding PVB binder. The effects of PVB on the shapes, sizes, distributions and flowability of polymer-ceramic composite powder agglomerates were investigated. There are several conclusions that can be drawn as shown below:

(1) The addition content of PVB has an optimal value (~3 wt. %). The modified SiC/PVB granules under the optimal addition of PVB exhibit good flowability and spreading performance.
(2) The resultant composite powder shows good laser absorptivity when the wavelength is below 500 nm, suggesting that SLS additive manufacturing of the composite powder may be applicable using 3D printers of the corresponding wavelength (e.g., below 500 nm).
(3) Results show that the addition of the polymer binder improves the size distribution characteristic and flowability of the granulated particles within a certain range. However, when the PVB content increases to a higher value (e.g., more than 7 wt. %), greater addition of PVB will not have much influence on the apparent density, tap density, Carr index or Hausner ratio.

## Figures and Tables

**Table 1:** Physical and mechanical properties of PVB material.

Material	*T_g_* (K)	Density (g/cm^3^)	Tensile Strength (MPa)	Modulus *G* (MPa)
PVB	289–350 [24]	1.08 [25]	10–17 [24]	0.3–0.8 [24]
